# Influence of Post Heat Treatment Condition on Corrosion Behavior of 18Ni300 Maraging Steel Manufactured by Laser Powder Bed Fusion

**DOI:** 10.3390/mi13111977

**Published:** 2022-11-15

**Authors:** Kichang Bae, Dongmin Shin, Jun-Ho Kim, Wookjin Lee, Ilguk Jo, Junghoon Lee

**Affiliations:** 1 Department of Metallurgical Engineering, Pukyong National University, Busan 48513, Republic of Korea; 2 Dongnam Division, Korea Institute of Industrial Technology, Yangsan 50623, Republic of Korea; 3 School of Materials Science and Engineering, Pusan National University, Busan 46241, Republic of Korea; 4 Department of Advanced Materials Engineering, Dong-Eui University, Busan 47340, Republic of Korea

**Keywords:** laser powder bed fusion, maraging steel, post heat treatment, corrosion behavior, anisotropy

## Abstract

Laser powder bed fusion (LPBF) is a promising additive-manufacturing process for metallic materials. It has the advantage of flexibility in product design, such that various mechanical parts can be fabricated. However, because metal parts are built-up in a layer-by-layer manner, the material fabricated by LPBF has an anisotropic microstructure, which is important for the design of materials. In this study, the corrosion resistance of 18Ni300 maraging steel (MS) fabricated by LPBF was explored considering the building direction. Furthermore, the effects of heat treatment and aging on the microstructure and corrosion resistance were investigated. Sub-grain cells formed by rapid cooling in LPBF improve the corrosion resistance of MS. As a result, the as-built MS has the highest corrosion resistance. However, the sub-grain cells are eliminated by heat treatment or aging, which causes the deterioration of corrosion resistance. In the case of 18Ni300 MS, the cylindrical sub-grain cells are formed and aligned along the heat dissipation direction, which is similar to the building direction; thus, a significant anisotropy in corrosion resistance is found in the as-built MS. However, such anisotropy in corrosion resistance is diminished by heat treatment and aging, which eliminates the sub-grain cells.

## 1. Introduction

Additive manufacturing (AM) is a fabrication process based on metal powder metallurgy. It is completely different from the conventional process, and it involves printing the desired parts layer-by-layer. These methods provide new possibilities for the production of final shaped components with a single process, reducing the unit time and production cost. During the AM process, materials undergo rapid melting and solidification (approximately 10^6–7^ K/s); therefore, unusual microstructures form that affect anisotropy of the mechanical and chemical properties [1,2,3,4,5]. Various energy sources (e.g., e-beam or laser) have been used to AM process for high resolution of product. Especially, the laser energy source is used for various processes and production of the various materials [6,7,8,9,10]. Metal AM processes, such as directed energy deposition (DED) technologies are mainly used to produce large-scale metallic AM structures with high deposition rates and to repair damaged components [11,12,13,14], while the powder bed fusion (PBF) process is applied to small and high-complexity parts [15,16,17,18,19].

Maraging steel (MS) is a low-carbon, high-alloy special steel with unique properties, such as high strength, high fracture toughness, excellent weldability, and excellent hardenability, dimensional stability, and simplicity of heat treatment. Its properties can be obtained by martensitic phase transformation during post-heat treatment. In addition, MS has a very low carbon content, which greatly reduces the risk of quench cracking, and it has excellent corrosion resistance owing to its high Ni content with no carbides. Because of these advantages, MS is emerging as an alloy that can replace high-carbon steel used in high-performance engineering fields, such as aerospace, military parts, transformation, tool and die industries, electromechanical properties, and motor racing parts (e.g., hot-work dies, bearing gear parts, drill chucks, and rocket motors case) [20,21,22]. Therefore, various grade MSs are generally in demand to apply for various industries. In particular, the low-carbon characteristic gives MS excellent weldability because carbide formation or carbon segregation is not a problem; the risk of quench cracking is sharply decreased, while the high nickel (Ni) and molybdenum (Mo) contents and lack of carbides provide superior corrosion resistance. Therefore, it is very advantageous to apply the AM process. Due to the good weldability of MS, the fabrication of MS by AM is a practicable task. It considers the high potential to replace tool and hardened steel alloys fabricated by traditional manufacturing methods used in a variety of applications [23,24,25,26].

Several studies have been conducted on the AM of MS, demonstrating various advantages. For example, Mutua et al. [27] studied the surface quality, relative density, microstructure, and hardness of MS according to the AM process variables and revealed the optimal process parameters, which provide good surface quality with a relative density of 99.8% and surface roughness (*R_a_*) of 35 μm. Casati et al. [28] investigated microstructure and tensile strength changes by performing various aging heat treatments on 18Ni300 MS manufactured by the selective laser melting (SLM) process. They reported that the reversal of martensite into the austenite phase was observed after aging heat treatment, and various aging treatment temperatures significantly improved the tensile strength while decreasing the elongation. Paul et al. [29] evaluated the changes in the mechanical properties of 18Ni300 MS manufactured by the PBF process owing to microstructure evolution and compared the results with those of MS manufactured by the casting process. Through post-heat treatment, they improved the yield strength to approximately 1.8 GPa and ultimate tensile strength to approximately 2.0 GPa. Lee et al. [30,31,32] examined microstructural changes and mechanical properties after performing various heat treatments on 18Ni300 MS manufactured by the PBF process. Such mechanical characteristics as hardness, tensile strength, and wear resistance showed anisotropy depending on the building direction in the as-built specimen; however, post heat treatment decreased the anisotropy. This research revealed the effects of microstructural changes, such as different melt pool geometries, decomposition of sub-grain cells, and fine precipitation, on mechanical properties during post heat treatment.

Research on the relationship between the mechanical properties and microstructure change of the additively manufactured MS according to the post heat treatment process has been conducted; however, previous studies have rarely focused on the corrosion properties of additively manufactured MS. In particular, the corrosion resistance of MS should be verified for the actual application of additively manufactured MS because of its high nickel, cobalt, and molybdenum contents, which improve corrosion resistance compared with the high-strength steel currently in use. In addition, because heat-treated MS also involves changes in the microstructure that cause changes in the mechanical properties, corrosion resistance needs to be evaluated.

In this study, the effects of various post-heat treatments on the corrosion resistance of MS manufactured by the laser powder bed fusion (LPBF) process in a seawater atmosphere were investigated in relation to the post-heat treatment condition with improved mechanical properties obtained in a previous study. The behavior of microstructural changes and corrosion resistance resulting from post-heat treatment were analyzed, and the corrosion resistance according to the building direction was studied by considering sides perpendicular to and horizontal to the building direction.

## 2. Materials and Methods

### 2.1. Preparation of Samples

Spherical gas-atomized 18Ni300 MS powder (OPM maraging, OPM Laboratory Co., Ltd., Kyoto, Japan) with an average particle size of −40 μm was used to produce the specimen used in this study. The chemical composition of the 18Ni300 MS powder used is shown in Table 1.

The specimen was produced using an LPBF-type metal 3D printer (OPM250L, Sodick Co., Ltd., Kyoto, Japan), and the LPBF process variables selected to produce the specimens are described in detail in Table 2. It was prepared in a nitrogen environment with the oxygen of 1% or less to prevent the specimen from being oxidized during the process. A 90°-rotational scanning strategy was used, i.e., the laser scanning lines were tilted by 90° between each layer. Further details of the material, including the microstructure, hardness, tensile properties, and wear behavior relative to the building direction, have been reported previously [30,31,32].

**Figure 1:**
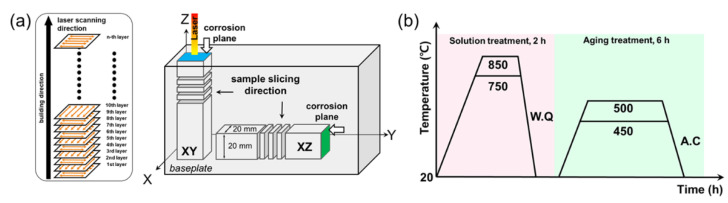
(**a**) Schematic diagram of sample geometries, scanning strategy, building direction, corrosion test planes, and (**b**) various heat treatment processes.

Two types of block with a size of 20 × 20 × 100 mm^3^ were printed to examine the anisotropy depending on the building direction while manufacturing the 18Ni300 MS specimen using the LPBF process. Each specimen was cut from the block to have a thickness of 3 mm. The corrosion surface was selected as a plane parallel to or perpendicular to the building direction, as shown in Figure 1a. The plane parallel to the building direction was named XZ (green area), and the plane perpendicular to the building direction was named XY (blue area). Three heat treatment conditions were selected to evaluate the corrosion resistance according to the post heat treatment. Each heat treatment condition was selected by referring to the results of a preliminary study on the improvement of the mechanical strength by post heat treatment and the relationship depending on the building direction in the as-built state [30,31,32]. Figure 1b and Table 3 show the various heat treatment conditions and specimen notations according to the heat treatment, respectively. All post heat treatments were performed using a box-type furnace in an air atmosphere. To prevent the oxidation of the specimen surface, each specimen was wrapped in a protective heat-treatment foil. After the solution treatment and aging heat treatment, water quenching (W.Q) and air cooling (A.C) were performed, respectively.

### 2.2. Material Characterizations

An optical microscopy (OM, ECLIPSE LV150N, Nikon, Tokyo, Japan) and field-emission scanning electron microscopy (FE-SEM, JSM-7200F, Jeol Inc., Tokyo, Japan) were used to observe the microstructure of the 18Ni300 MS specimen prepared by the LPBF process. Modified Fry’s regent, 1 g of CuCl_2_, 25 mL of HNO_3_, 50 mL of HCl, and 150 mL of distilled water were used as the etchant.

X-ray diffractometer diffraction (XRD, EMPYREAN, PANalytical BV, Almelo, Netherlands) was used to analyze the crystal structure of sample. Cu Kα (λ = 1.5406 Å) monochromatic beam generated with 40 kV and 40 mA was scanned from 20° to 80° with a scanning speed of 1.5 °/min.

Potentiodynamic polarization and electrochemical impedance spectroscopy (EIS) were conducted in 3.5 wt.% NaCl solution at room temperature using a flat cell with Ag/AgCl (saturated KCl, reference electrode) and platinum mesh (counter electrode) and potentiostat (VersaSTAT3, AMETEK, Berwyn, PA, USA). As for potentiodynamic polarization, the initial and final potential for scan (2 mV/s) were −300 and 1500 mV versus open circuit potential (OCP), which is stabilized by immersing sample in 3.5 wt.% NaCl solution for 20 min. As for EIS, AC signal with an amplitude of 10 mV vs. OCP was applied with the frequency range from 10 kHz to 0.1 Hz. The specimen, which was subjected to a potentiodynamic polarization test to observe the corrosion surface using FE-SEM, was ultrasonically washed with 0.1 M H_2_SO_4_ for approximately 10–20 s to remove the corrosion products. To minimize the experimental error in electrochemical corrosion analysis (i.e., potentiodynamic polarization and EIS), we used seven samples fabricated by each condition. Excluding potentiodynamic polarization curves with maximum and minimum corrosion current density, five corrosion current densities were averaged. In case of EIS, after model fitting of seven results, an averaged charge transfer resistance was estimated excluding the maximum and minimum value. Then, a potentiodynamic polarization and EIS curves, which have the most similar values (i.e., corrosion current density and charge transfer resistance) to average, were shown as representative results.

## 3. Results and Discussion

**Figure 2:**
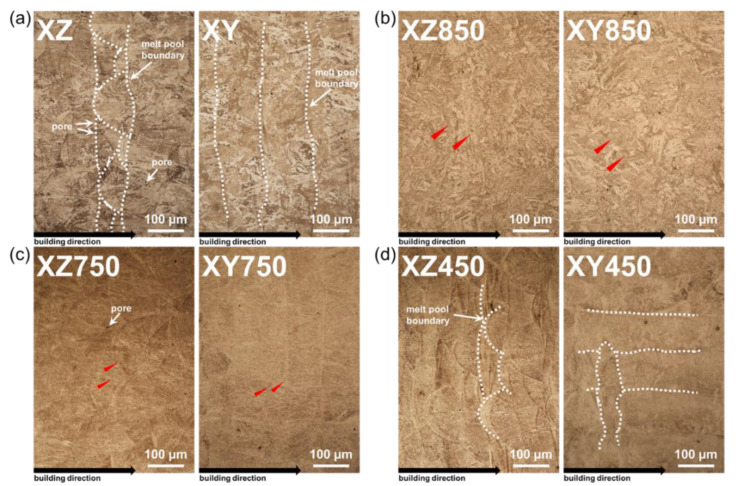
OM images of laser-powder-bed-fused MS: (**a**) as-built, (**b**) 850 °C heat treated, (**c**) 750 °C heat treated, and (**d**) 450 °C heat treated.

Figure 2 shows OM images of the microstructural changes according to various post-heat treatments of 18Ni300 maraging steel (MS) manufactured by the LPBF process. Figure 2a shows the microstructure of the as-built state specimen, and the melt-pool and melt-pool boundary formation during the LPBF process can be observed (white dotted line). Furthermore, pores were observed near the melt-pool boundary. This is a defect that can occur in the printing process because of the interspace present in the different sizes of metal powders in the melting process of 18Ni300 steel powders by the laser [33,34]. Figure 2b shows the microstructure of the specimen, which was solution treated at 850 °C for 2 h and then aged at 500 °C for 6 h. Unlike the as-built state specimen, the melt-pool and melt-pool boundaries cannot be completely observed. The melt-pool and melt-pool boundaries were rarely observed in specimen solution treated for 2 h at 750 °C and then aged for 6 h at 450 °C (see Figure 2c). However, the melt-pool and melt-pool boundaries can clearly be observed in specimens aged for 6 h at 450 °C without solution treatment (see Figure 2d). This result indicates that the melt-pool of 18Ni300 MS manufactured by the LPBF process disappeared during the solution treatment process. In the OM image, a black phase can be observed regardless of the as-built state and post-heat-treatment conditions (red triangle). 

**Figure 3:**
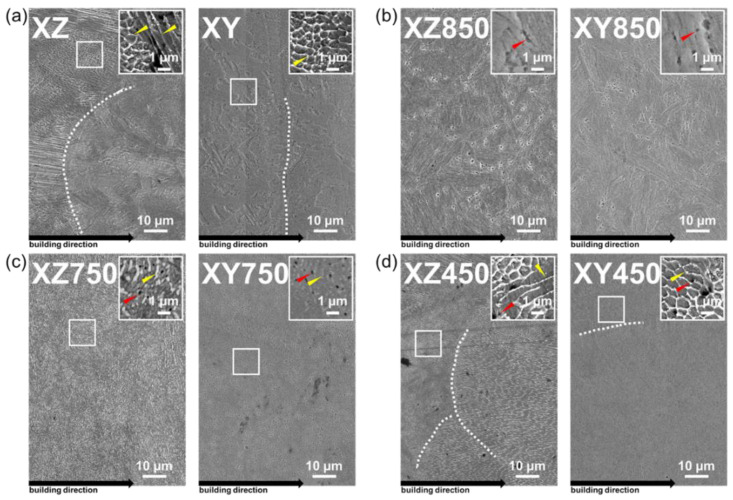
SEM images of XZ-plane of laser-powder-bed-fused MS: (**a**) as-built, (**b**) 850 °C heat-treated, (**c**) 750 °C heat-treated, and (**d**) 450 °C heat-treated.

To observe in detail the phenomenon where the melt-pool disappears and the black phase depends on the heat treatment conditions, the microstructure was observed at high magnification using FE-SEM, and the results are shown in Figure 3.

In Figure 3a, in the as-built states, the XZ and XY specimens, melt-pool and melt-pool boundaries, and sub-grain cells with a size of approximately 1 μm or less can be observed. Sub-grain cells are distinct microstructures created by fast cooling in the printing process; in particular, the formation of sub-grain cells with a size of 2 μm or less has been reported to increase the mechanical strength [35,36,37]. Such sub-grain cells may disappear by diffusion during the post-heat treatment process. Figure 3b,d,e show that the shape of the sub-grain cell changes or disappears owing to diffusion depending on the heat treatment conditions. Under 850 °C heat treatment condition, the sub-grain cell disappeared completely and had an entirely different microstructure from the as-built specimen. However, sub-grain cells were still observed in 750 °C and 450 °C heat treatments. The sub-grain cell was decomposed by diffusion during the heat treatment of XZ750 and XY750; however, the sub-grain cell was not completely decomposed, and the network collapsed (see Figure 3c). In contrast, in the XZ450 and XY450 specimens, a sub-grain cell shape similar to that of the as-built state was observed (see Figure 3d). This reveals that the sub-grain cell was not completely decomposed by the diffusion process under 450 °C directly aged heat treatment conditions; however, the sub-grain cell network weakened. In other words, the network almost disappeared or was completely decomposed in the post-heat treatment conditions followed by the solution treatment of MS manufactured by the LPBF process. The black phase observed in the FE-SEM image is a titanium-rich precipitate generated by the post-heat treatment process. As a result of the EDS mapping in a previous study, in which a microstructure analysis of the same specimen used in this study was conducted, it was found that the precipitate did not contain any other metallic elements or carbon, except for a high concentration of Ti [32]. Kang et al. [38] reported the presence of Ti-rich regions along the melt-pool boundary of MS manufactured by the SLM process. The precipitate changed its morphology into a spherical shape owing to the post-heat treatment. In FE-SEM observations, lath-type fine precipitates were observed around these spherical precipitates, and XRD analysis was performed to analyze them.

**Figure 4:**
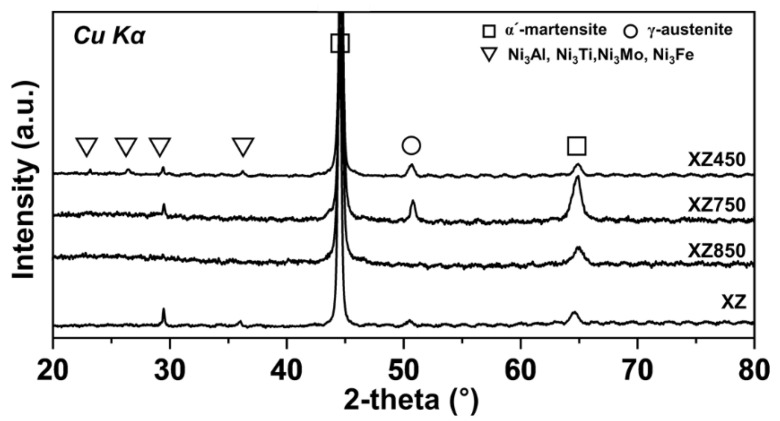
X-ray diffractometer diffraction (XRD) patterns of as-built state and heat-treated 18Ni300 MS.

Figure 4 shows the XRD analysis results of the as-built and post-heat-treated XZ specimens. The main diffraction peak is the α′-martensite phase in all specimens, which is formed by a rapid cooling rate of more than 10^6^ K/s during the LPBF manufacturing process and is almost fully martensitic phase in the as-built state. The lath-type precipitate observed corresponds to Ni-based intermetallic compounds, such as Ni_3_Al, Ni_3_Ti, Ni_3_Mo, and Ni_3_Fe, found in the range of 20°–40°. It is somewhat clearly observed in the XZ and XZ450 specimens and seen at approximately 28° in XZ750 specimen. No peaks of the Ti-rich phase were observed owing to its small fraction. Meanwhile, the retained γ-austenite phase was observed only in the XZ, XZ750, and XZ450 specimens but not in the XZ850 specimen. It is concluded that the retained γ-austenite phase completely disappeared during the solution heat treatment at 850 °C.

**Figure 5:**
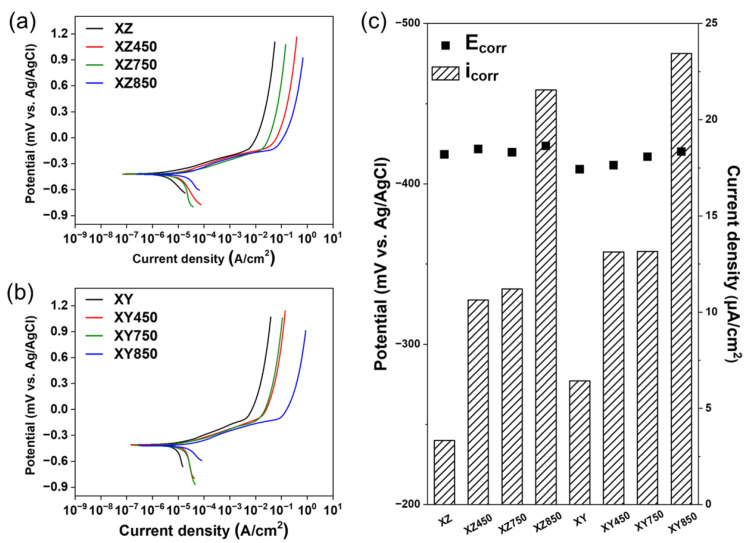
Corrosion resistance of each specimen in 3.5 wt.% NaCl solution: (**a**) potentiodynamic polarization curves of XZ-plane, (**b**) potentiodynamic polarization curves of XY-plane, and (**c**) comparison of corrosion potential (E_corr_) and corrosion current density (i_corr_).

Figure 5a,b show the potentiodynamic polarization curves of the sample in 3.5 wt.% NaCl solution. The corrosion current density and potential estimated by Tafel fitting are summarized in Figure 5c. For the as-built MS in the XZ-plane, the corrosion current density was 3.34 μA/cm^2^. The aging at 450 °C (XZ450) and heat treatment at 750 °C (XZ750) caused a partial dissolution of sub-grain cells in the as-built MS, which increased the corrosion current density to 10.63 and 11.21 μA/cm^2^, respectively. The wall of the sub-grain cell is formed by locally segregated Ni and Mo [39,40,41,42,43], which improve the corrosion resistance of steel by forming a passive layer; thus, the partial elimination of sub-grain cells decreases the corrosion resistance. Because of the complete elimination of sub-grain cells by heat treatment at 850 °C (XZ850), the corrosion current density increases significantly to 21.55 μA/cm^2^, which is 18.21 μA/cm^2^ higher than that of XZ. The change in the corrosion resistance of the as-built XY-plane upon heat treatment is similar to that in the XZ-plane. The corrosion current density of the XY-plane of the as-built MS is 6.43 μA/cm^2^. The corrosion current densities increased to 13.13 and 13.15 μA/cm^2^ after heat treatment at 750 °C (XY750) and aging at 450 °C (XY450), respectively, which partially eliminates the sub-grain cells of the as-built MS. In addition, the heat treatment at 850 °C completely eliminated the sub-grain cells, which significantly increased the corrosion current density to 23.45 μA/cm^2^ (17.02 μA/cm^2^ fold higher than XY). The corrosion current densities of XZ450 and XY450 are slightly lower than those of XZ750 and XY750, respectively. This indicates that the heat treatment at 750 °C is more effective for dissolving partial segregation of alloying elements than aging at 450 °C.

**Figure 6:**
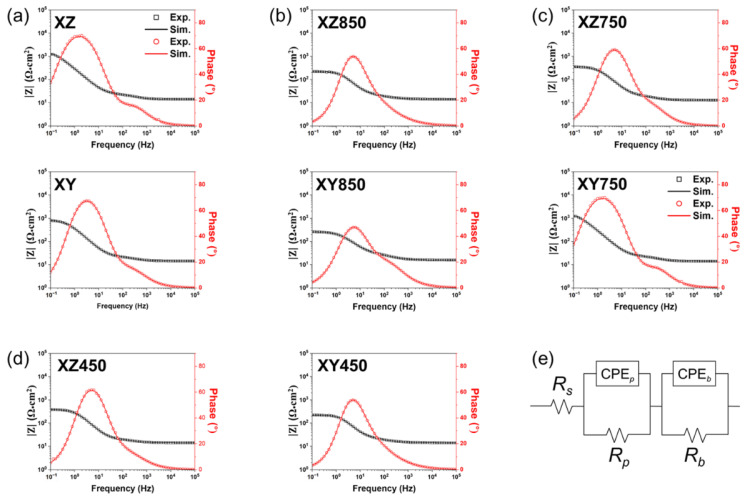
Bode spectra for each specimen in 3.5 wt.% NaCl solution: (**a**) as-built, (**b**) 850 °C heat treated, (**c**) 750 °C heat treated, and (**d**) 450 °C heat treated. (**e**) Equivalent circuit for data fitting.

In addition to the potentiodynamic polarization tests, the surface corrosion reactions of the laser-powder-bed-fused MSs were analyzed by EIS (Figure 6). An equivalent circuit for data analysis is shown in Figure 6e. 

In the equivalent circuit, *R_s_*, *R_p_*, and *R_b_* are the resistance of the electrolyte, porous surface oxide layer, and charge transfer reaction at the metal–oxide interface for corrosion, respectively. CPE*_p_* and CPE*_b_* are the capacitance of the double layer on the oxide surface and the barrier oxide layer, respectively. Furthermore, *n_p_* and *n_b_* correspond to the exponents of CPE*_p_* and CPE*_b_*, respectively. Each value in the equivalent circuit as a result of the model fitting is summarized in Table 4. 

Generally, *R_p_* + *R_b_* corresponds to corrosion resistance. However, no significant difference in the resistance for the surface oxide layer (*R_p_*) of MS according to the heat treatment and plane was identified, whereas clear differences in the charge transfer resistance were observed. In addition, *R_p_* is much lower than *R_b_*. Therefore, the corrosion resistance can be determined by the *R_b_*. The XZ-plane of the as-built MS showed the highest *R_b_*, which was significantly decreased by heat treatment or aging. And, samples heat treated at 850 °C, which completely eliminated the sub-cell structure, had the lowest *R_b_* among the tested XZ-planes (XZ, XZ750, XZ850, and XZ450). Similar to the XZ-plane, *R_b_* in the XY-plane also decreases by heat treatment or aging. These results also indicate that the corrosion resistance of the as-built MS is decreased by the dissolution of partially segregated sub-grain cells at high temperatures [44]. Moreover, these results agree well with the results of the potentiodynamic polarization test. Therefore, even though heat treatment improves the mechanical properties of laser-powder-bed-fused MSs, it should be noted that the corrosion resistance is determined by post-treatment.

Meanwhile, comparing the corrosion current density and charge transfer resistance of the XZ- and XY-planes reveals that the anisotropy in the corrosion resistance was similar to the mechanical properties [32]. The XZ-plane had a lower corrosion current density and higher charge transfer resistance than the XY-plane, even after heat treatment or aging. For as-built MS, the corrosion current density of XZ was lower than that of XY by 51%, and the charge transfer resistance of XZ was higher than that of XY by 46%. However, the anisotropy in corrosion resistance showed a lower corrosion current density and higher charge transfer resistance of the XZ-plane than the XY-plane, which was reduced by heat treatment and aging. In particular, after the heat treatment at 850 °C, the difference in corrosion current density and charge transfer resistance decreased to only 87% and 76%, respectively. Such anisotropy in corrosion resistance of laser-powder-bed-fused MS can be explained by the shape and arrangement of the sub-grain cells.

**Figure 7:**
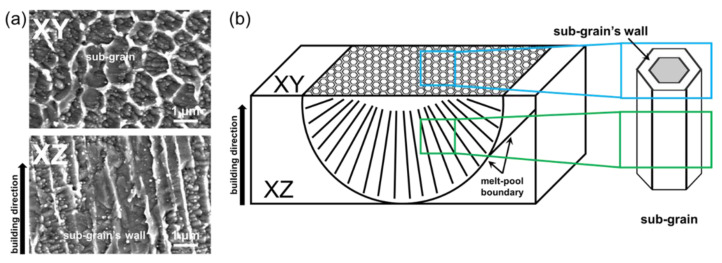
(**a**) Microstructures of the XZ and XY and (**b**) schematic illustrations of the sub-grain structure in a single melt track.

Figure 7 shows a high-magnification microstructure image of the as-built MS of the XZ and XY. The sub-grain cell is formed by rapid cooling along the heat transfer direction; thus, the local segregation of the sub-grain cell is aligned with the melt pool boundary. Therefore, cylindrical sub-grain cells with a high-aspect-ratio aligned from the melt-pool boundary to the melt-pool surface can be observed on the XZ-plane (see Figure 7a). In contrast, the cylindrical sub-grain cell is formed toward the melt pool surface; thus, the cylindrical shape of the sub-grain cell cannot be observed on the XY-plane (see Figure 7a). In addition, alloying elements of Ni and Mo, which improve the corrosion resistance of steel, are segregated to form a sub-grain cell wall. Therefore, the wall of the sub-grain cell has higher corrosion resistance than that inside the cell. In the case of the XZ-plane, the wall of the sub-grain cell is exposed to the outside such that the wall can act as a barrier to inhibit the propagation of corrosion toward the inside of the sub-grain cell (see Figure 7b). In contrast, the inside of the cylindrical sub-grain cell is exposed to the outside of the XY-plane; thus, the wall cannot inhibit the propagation of corrosion through the inside of the sub-grain cell. Therefore, the XZ-plane of the as-built maraging streel exhibits higher corrosion than the XY-plane. However, the compositional segregation between the sub-grain cell inside and the wall is homogenized by heat treatment or aging, promoting the diffusion of alloying elements, and the sub-grain cell can be eliminated. Therefore, the anisotropy of as-built MS in corrosion resistance can be reduced by heat treatment or aging, such that laser-powder-bed-fused MS with a completely homogenized microstructure has isotropy in corrosion resistance.

**Figure 8:**
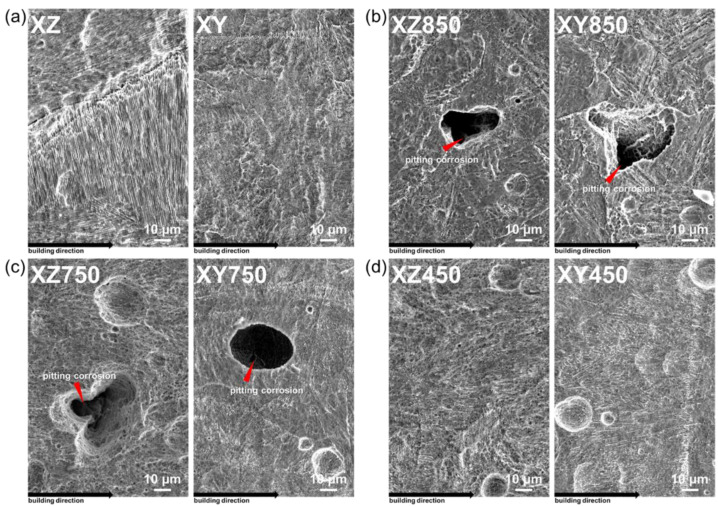
Corrosion surfaces of each specimen after potentiodynamic polarization test in 3.5 wt.% NaCl solution. (**a**) as-built, (**b**) 850 °C heat treated, (**c**) 750 °C heat treated, and (**d**) 450 °C heat treated.

The changes in the microstructure of laser-powder-bed-fused MS affect not only the corrosion resistance, but also the corrosion behavior. Figure 8 shows the surface of the sample after the potentiodynamic polarization test. The surfaces of the as-built (XZ and ZY) and aged (XZ450 and XY450) samples were similar, showing uniform corrosion. In addition, no differences in the corroded surface due to the testing plane and direction of the cylindrical sub-grain cell were identified. However, significant pits on the surface of the samples subjected to heat treatment (XZ750, XZ850, XY750, and XY850) were observed, indicating that localized pitting can occur with uniform corrosion. In the case of heat treatment at 750 °C or 850 °C, a Ti-rich phase was precipitated in the grains [32]. The Ti-rich phase can act as a cathode in the galvanic couple with the MS matrix such that the Ti-rich phase causes a potential difference around the precipitates. Such a significant unbalance in the surface potential caused by precipitation can be attributed to the occurrence of pitting corrosion. Therefore, even though the heat treatment of laser-powder-bed-fused MS improves the mechanical properties (i.e., yield strength, tensile strength, and wear resistance), it should be noted that the post-heat treatment eliminates sub-grain cells and causes precipitation of the Ti-rich phase, which not only causes the corrosion resistance to deteriorate but also increases the risk of pitting corrosion.

## 4. Conclusions

In this work, the corrosion resistance of 18Ni300 MS fabricated via LPBF was studied. The corrosion resistance related to the building direction and the influence of post heat treatment conditions were analyzed. The main findings can be summarized as follows:

1. LPBF of 18Ni300 MS creates a distinct microstructure with aligned cylindrical sub-grain cells owing to its rapid solidification. The wall of the sub-grain cell is formed by the segregation of alloying elements, such as Ni and Mo, which improve the corrosion resistance of steel. Because of this sub-grain cell, the as-built 18Ni300 MS has high corrosion resistance.
2. However, heat treatment and aging, which eliminate the sub-grain cell, cause the corrosion resistance to deteriorate. In particular, the heat treatment at 850 °C, which completely eliminates the sub-grain cell and forms Ti-rich precipitates, significantly reduces the corrosion resistance of the as-built 18Ni300 MS.
3. Owing to the alignment of the cylindrical sub-grain cell, a significant anisotropy in the corrosion resistance of 18Ni300 MS occurs according to the building direction. However, such anisotropy in corrosion resistance is also diminished by heat treatment and aging because the cylindrical sub-grain cell is eliminated.
4. Heat treatment and aging to improve the mechanical properties of 18Ni300 MS fabricated by LPBF should be designed considering that the process can cause corrosion resistance to deteriorate.

## Figures and Tables

**Table 1:** Chemical compositions of 18Ni300 MS powder.

Element	Fe	Ni	Co	Mo	Ti	Mn	Al
wt.%	Bal.	17.0–19.0	8.5	4.0	0.7	≤0.1	≤0.1

**Table 2:** Parameters used in the LPBF process.

Parameter	Setting
Laser power (W)	420
Scanning speed (mm/s)	1000
Hatch spacing (mm)	0.1
Lamination thickness (mm)	0.04

**Table 3:** Sample notation according to the post-heat treatment condition.

Sample Name	Solution Treatment Temperature (°C)	Time (h)	Aging Treatment Temperature (°C)	Time (h)
XZ	-	-	-	-
XY	-	-	-	-
XZ850	850	2	500	6
XY850	850	2	500	6
XZ750	750	2	450	6
XY750	750	2	450	6
XZ450	-	-	450	6
XY450	-	-	450	6

**Table 4:** Fitted data for parameter of equivalent circuit.

Sample Name	*R_s_*, Ω cm^2^	CPE*_p_*, mF/cm^2^	*n*_p_	*R_p_*, Ω cm^2^	CPE*_b_*, mF/cm^2^	*n_b_*	*R_b_*, Ωcm^2^
XZ	14.12	0.64	0.88	8.04	0.18	0.91	1743.00
XY	14.37	0.44	0.84	7.29	0.29	0.95	808.90
XZ850	15.83	0.55	0.78	13.28	0.42	0.90	234.90
XY850	13.99	2.15	0.65	11.94	0.53	0.99	195.30
XZ750	15.10	0.31	0.79	12.57	0.24	0.99	322.30
XY750	12.99	0.51	0.84	6.85	0.45	0.94	342.50
XZ450	14.10	0.43	0.79	5.97	0.56	0.96	364.80
XY450	13.97	0.34	0.65	16.81	1.53	0.99	325.30

## References

[1] Ngo T.D., Kashani A., Imbalzano G., Nguyen K.T.Q., Hui D. (2018). Additive manufacturing (3D printing): A review of materials, methods, applications and challenges. Compos. Part B Eng..

[2] DebRoy T., Wei H.L., Zuback J.S., Mukherjee T., Elmer J.W., Milewski J.O., Beese A.M., Wilson-Heid A., De A., Zhang W. (2018). Additive manufacturing of metallic components—Process, structure and properties. Prog. Mater. Sci..

[3] Tofail S.A.M., Koumoulos E.P., Bandyopadhyay A., Bose S., O’Donoghue L., Charitidis C. (2018). Additive manufacturing: Scientific and technological challenges, market uptake and opportunities. Mater. Today.

[4] Prakash K.S., Nancharaih T., Rao V.V.S. (2018). Additive Manufacturing Techniques in Manufacturing—An Overview. Mater. Today Proc..

[5] Maamoun A.H., Elbestawi M., Dosbaeva G.K., Veldhuis S.C. (2018). Thermal post-processing of AlSi10Mg parts produced by Selective Laser Melting using recycled powder. Addit. Manuf..

[6] You R., Liu Y.Q., Hao Y.L., Han D.D., Zhang Y.L., You Z. (2020). Laser Fabrication of Graphene-Based Flexible Electronics. Adv. Mater..

[7] Fu X.-Y., Chen Z.-D., Han D.-D., Zhang Y.-L., Xia H., Sun H.-B. (2020). Laser fabrication of graphene-based supercapacitors. Photonics Res..

[8] Ye X., Lin Z., Zhang H., Zhu H., Liu Z., Zhong M. (2015). Protecting carbon steel from corrosion by laser in situ grown graphene films. Carbon.

[9] Jiba Z., Focke W.W., Kalombo L., Madito M.J. (2020). Coating processes towards selective laser sintering of energetic material composites. Def. Technol..

[10] Yi J., Zhou H., Wei W.H., Han X.C., Han D.D., Gao B.R. (2021). Micro-/Nano-Structures Fabricated by Laser Technologies for Optoelectronic Devices. Front. Chem..

[11] Park Y.K., Ha K., Bae K.C., Shin K.Y., Lee K.Y., Shim D.-S., Lee W. (2022). Mechanical properties and wear resistance of direct energy deposited Fe–12Mn–5Cr–1Ni-0.4C steel deposited on spheroidal graphite cast iron. J. Mater. Res. Technol..

[12] Park Y.K., Ha K., Shin K.Y., Lee K.Y., Kim D.J., Kwon S.-H., Lee W. (2021). Wear resistance of direct-energy–deposited AISI M2 tool steel with and without post-heat treatment. Int. J. Adv. Manuf. Technol..

[13] Linnenbrink S., Alkhayat M., Pirch N., Gasser A., Schleifenbaum H. (2021). DED for repair and manufacture of turbomachinery components. 3D Printing for Energy Applications.

[14] Svetlizky D., Das M., Zheng B., Vyatskikh A.L., Bose S., Bandyopadhyay A., Schoenung J.M., Lavernia E.J., Eliaz N. (2021). Directed energy deposition (DED) additive manufacturing: Physical characteristics, defects, challenges and applications. Mater. Today.

[15] Narasimharaju S.R., Zeng W., See T.L., Zhu Z., Scott P., Jiang X., Lou S. (2022). A comprehensive review on laser powder bed fusion of steels: Processing, microstructure, defects and control methods, mechanical properties, current challenges and future trends. J. Manuf. Process..

[16] Cobbinah P.V., Nzeukou R.A., Onawale O.T., Matizamhuka W.R. (2020). Laser Powder Bed Fusion of Potential Superalloys: A Review. Metals.

[17] Khorasani A., Gibson I., Veetil J.K., Ghasemi A.H. (2020). A review of technological improvements in laser-based powder bed fusion of metal printers. Int. J. Adv. Manuf. Technol..

[18] Singh R., Gupta A., Tripathi O., Srivastava S., Singh B., Awasthi A., Rajput S.K., Sonia P., Singhal P., Saxena K.K. (2020). Powder bed fusion process in additive manufacturing: An overview. Mater. Today Proc..

[19] King W.E., Anderson A.T., Ferencz R.M., Hodge N.E., Kamath C., Khairallah S.A., Rubenchik A.M. (2015). Laser powder bed fusion additive manufacturing of metals; physics, computational, and materials challenges. Appl. Phys. Rev..

[20] Zhu F., Yin Y.F., Faulkner R.G. (2014). Microstructural control of maraging steel C300. Mater. Sci. Technol..

[21] Vander Voort G.F., Lucas G.M., Manilova E.P. (2004). Metallography and microstructures of stainless steels and maraging steels. ASM Handbook.

[22] Kapoor R., Kumar L., Batra I.S. (2003). A dilatometric study of the continuous heating transformations in 18 wt.% Ni maraging steel of grade 350. Mater. Sci. Eng. A.

[23] Krol M., Snopinski P., Hajnys J., Pagac M., Lukowiec D. (2020). Selective Laser Melting of 18NI-300 Maraging Steel. Materials.

[24] Özer G., Karaaslan A. (2020). A Study on the Effects of Different Heat-Treatment Parameters on Microstructure–Mechanical Properties and Corrosion Behavior of Maraging Steel Produced by Direct Metal Laser Sintering. Steel Res. Int..

[25] Turk C., Zunko H., Aumayr C., Leitner H., Kapp M. (2019). Advances in Maraging Steels for Additive Manufacturing. BHM Berg-Und Hüttenmännische Mon..

[26] Campanelli S.L., Contuzzi N., Posa P., Angelastro A. (2019). Study of the aging treatment on selective laser melted maraging 300 steel. Mater. Res. Express.

[27] Mutua J., Nakata S., Onda T., Chen Z.-C. (2018). Optimization of selective laser melting parameters and influence of post heat treatment on microstructure and mechanical properties of maraging steel. Mater. Des..

[28] Casati R., Lemke J., Tuissi A., Vedani M. (2016). Aging Behaviour and Mechanical Performance of 18-Ni 300 Steel Processed by Selective Laser Melting. Metals.

[29] Paul M.J., Muniandy Y., Kruzic J.J., Ramamurty U., Gludovatz B. (2022). Effect of heat treatment on the strength and fracture resistance of a laser powder bed fusion-processed 18Ni-300 maraging steel. Mater. Sci. Eng. A.

[30] Chang Bae K., Kim D., Kim Y.H., Oak J.-J., Lee H., Lee W., Park Y.H. (2021). Effect of heat treatment, building direction, and sliding velocity on wear behavior of selectively laser-melted maraging 18Ni-300 steel against bearing steel. Wear.

[31] Bae K., Kim D., Lee W., Park Y. (2021). Wear Behavior of Conventionally and Directly Aged Maraging 18Ni-300 Steel Produced by Laser Powder Bed Fusion. Materials.

[32] Kim D., Kim T., Ha K., Oak J.-J., Jeon J.B., Park Y., Lee W. (2020). Effect of Heat Treatment Condition on Microstructural and Mechanical Anisotropies of Selective Laser Melted Maraging 18Ni-300 Steel. Metals.

[33] Rao B.S., Rao T.B. (2022). Effect of Process Parameters on Powder Bed Fusion Maraging Steel 300: A Review. Lasers Manuf. Mater. Process..

[34] Demir A.G., Previtali B. (2017). Investigation of remelting and preheating in SLM of 18Ni300 maraging steel as corrective and preventive measures for porosity reduction. Int. J. Adv. Manuf. Technol..

[35] Ma M., Wang Z., Zeng X. (2017). A comparison on metallurgical behaviors of 316L stainless steel by selective laser melting and laser cladding deposition. Mater. Sci. Eng. A.

[36] Segura I.A., Mireles J., Bermudez D., Terrazas C.A., Murr L.E., Li K., Injeti V.S.Y., Misra R.D.K., Wicker R.B. (2018). Characterization and mechanical properties of cladded stainless steel 316L with nuclear applications fabricated using electron beam melting. J. Nucl. Mater..

[37] Wanni J., Michopoulos J.G., Achuthan A. (2022). Influence of cellular subgrain feature on mechanical deformation and properties of directed energy deposited stainless steel 316 L. Addit. Manuf..

[38] Kang N., Ma W., Heraud L., El Mansori M., Li F., Liu M., Liao H. (2018). Selective laser melting of tungsten carbide reinforced maraging steel composite. Addit. Manuf..

[39] Revilla R.I., Van Calster M., Raes M., Arroud G., Andreatta F., Pyl L., Guillaume P., De Graeve I. (2020). Microstructure and corrosion behavior of 316L stainless steel prepared using different additive manufacturing methods: A comparative study bringing insights into the impact of microstructure on their passivity. Corros. Sci..

[40] Saeidi K., Gao X., Zhong Y., Shen Z.J. (2015). Hardened austenite steel with columnar sub-grain structure formed by laser melting. Mater. Sci. Eng. A.

[41] Ziętala M., Durejko T., Polański M., Kunce I., Płociński T., Zieliński W., Łazińska M., Stępniowski W., Czujko T., Kurzydłowski K.J. (2016). The microstructure, mechanical properties and corrosion resistance of 316L stainless steel fabricated using laser engineered net shaping. Mater. Sci. Eng. A.

[42] Haghdadi N., Ledermueller C., Chen H., Chen Z., Liu Q., Li X., Rohrer G., Liao X., Ringer S., Primig S. (2022). Evolution of microstructure and mechanical properties in 2205 duplex stainless steels during additive manufacturing and heat treatment. Mater. Sci. Eng. A.

[43] Wang Y.M., Voisin T., McKeown J.T., Ye J., Calta N.P., Li Z., Zeng Z., Zhang Y., Chen W., Roehling T.T. (2018). Additively manufactured hierarchical stainless steels with high strength and ductility. Nat. Mater..

[44] Bae K., Shin D., Lee J., Kim S., Lee W., Jo I., Lee J. (2022). Corrosion Resistance of Laser Powder Bed Fused AISI 316L Stainless Steel and Effect of Direct Annealing. Materials.

